# Effect of moderately increased thyroid‐stimulating hormone levels and presence of thyroid antibodies on pregnancy among infertile women

**DOI:** 10.1002/rmb2.12306

**Published:** 2019-11-11

**Authors:** Shuhei So, Wakasa Yamaguchi, Nao Murabayashi, Naomi Miyano, Fumiko Tawara

**Affiliations:** ^1^ Department of Reproductive and Perinatal Medicine Hamamatsu University School of Medicine Hamamatsu‐Shi Shizuoka Japan; ^2^ Tawara IVF Clinic Shizuoka Japan

**Keywords:** assisted reproductive technology, cumulative pregnancy rate, subclinical hypothyroidism, thyroid antibody, thyroid‐stimulating hormone

## Abstract

**Purpose:**

To study the effects of mildly elevated thyroid‐stimulating hormone (TSH) levels and thyroid antibodies on pregnancy rates among infertile women and their potential contribution to prolonged infertility treatment.

**Methods:**

This case‐control study included 1479 women who underwent infertility treatment between March 2015 and August 2017. Cumulative pregnancy and miscarriage rates after assisted reproductive technology (ART) or non‐ART treatments were compared between women with TSH <2.5 mIU/L and those with TSH 2.5‐3.5 mIU/L and between women with and without thyroid antibody positivity.

**Results:**

The cumulative pregnancy rate of women with TSH 2.5‐3.5 mIU/L was similar to that of women with TSH <2.5 mIU/L in the non‐ART (hazard ratio [HR], 0.85; 95% confidence interval [CI], 0.56‐1.23) and ART (HR, 1.17; 95% CI, 0.93‐1.47) groups. Thyroglobulin antibody (TgAb) and thyroid peroxidase antibody (TPOAb) had no correlation with cumulative pregnancy rates. In the non‐ART and ART groups, HRs for TgAb were 0.87 (95% CI, 0.55‐1.32) and 1.09 (95% CI, 0.84‐1.39) and HRs for TPOAb were 0.88 (95% CI, 0.52‐1.39) and 1.29 (95% CI, 0.97‐1.68), respectively.

**Conclusions:**

Cumulative pregnancy rates and miscarriage rates were similar between women with TSH <2.5 mIU/L and those with TSH 2.5‐3.5 mIU/L and were independent of thyroid antibody positivity.

## 1 INTRODUCTION

Thyroid function and reproductive function are closely related.^1^ For example, hyper‐ and hypothyroidism are associated with amenorrhea.^1^ Therefore, thyroid dysfunction is considered a cause of infertility.^1^ Subclinical hypothyroidism (SCH) is defined as high levels of serum thyroid‐stimulating hormone (TSH) associated with normal free thyroxine (FT4). Thyroid antibodies, including thyroid peroxidase antibody (TPOAb) and thyroglobulin antibody (TgAb), are known risk factors for SCH.^2^ SCH is also reported to be associated with infertility.^3^, ^4^, ^5^, ^6^ Furthermore, the presence of SCH may induce maternal and fetal complications during pregnancy, such as miscarriage, hypertensive disorders of pregnancy, and decreased intelligence quotient of children.^7^, ^8^, ^9^ Therefore, management of thyroid function in women who plan to become pregnant may improve their outcomes and those of their children. However, studies have used different cutoff points of TSH to define SCH. Several investigators have claimed that TSH levels should be controlled and adjusted to a target level of 2.5 mIU/L among women seeking to become pregnant.^3^, ^4^, ^5^, ^6^, ^10^ Relying on a TSH cutoff value of 2.5 mIU/L is debatable.^11^, ^12^, ^13^, ^14^ TSH levels vary according to measurement method, age, and ethnicity.^15^ Therefore, each laboratory should set its standards based on the patient's background and measurement method. Furthermore, in most studies, the pregnancy rate used to assess the reproductive performance of infertile women with SCH has been reported in terms of pregnancy per cycle. However, the pregnancy rate per cycle does not account for the fact that the period for becoming pregnant could be shortened if the cutoff value of TSH is 2.5 mIU/L. Therefore, in this study, we examined whether moderately increased TSH and the presence of thyroid antibodies affected the cumulative pregnancy rate of women seeking to become pregnant. The cumulative pregnancy rate used to assess reproductive performance provides a better estimate of the history of multiple infertility treatments.

## 2 MATERIALS AND METHODS

### 2.1 Subjects

**Figure 1:**
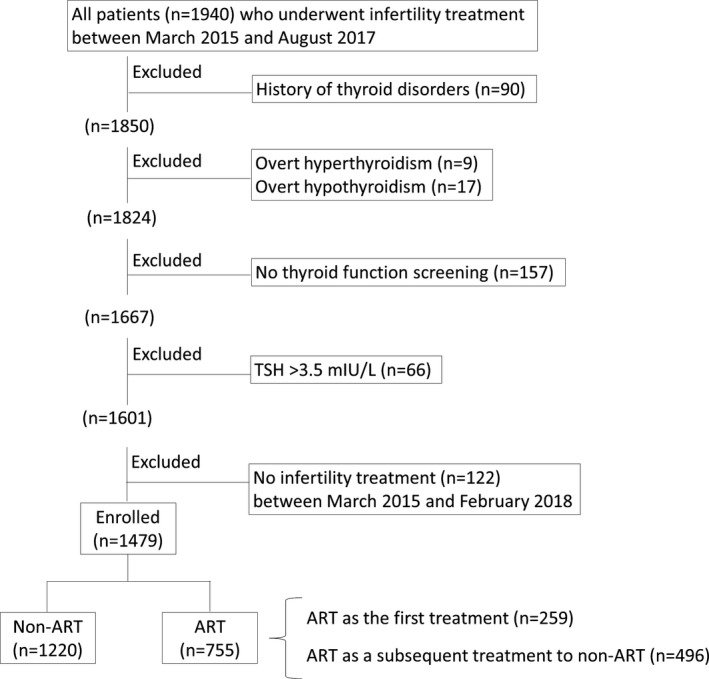
Flow diagram of study subjects

Between March 2015 and August 2017, 1940 infertile women who visited the Tawara IVF Clinic (Shizuoka, Japan) for the first time were recruited for this case‐controlled study. The study was approved by the Institutional Review Board (IRB) at Tawara IVF Clinic and Hamamatsu University School of Medicine. Clinical information was collected by reviewing medical records after opt‐out recruitment conducted during this study. A flowchart of subjects included in the study is shown in Figure 1. Of the 1940 women recruited, 90 with a history of abnormal thyroid function, including Graves' disease or Hashimoto disease, were excluded from this study. Of the remaining 1850 women, 9 and 17 who were diagnosed with overt hyperthyroidism and overt hypothyroidism, respectively, during the screening examination were also excluded. Of the remaining 1824 patients, 157 who underwent TSH assessment at other clinics were excluded because detailed information on aspects such as the TSH measurement method could not be retrieved. Tawara IVF Clinic manages patients with TSH >3.5 mIU/L, with cutoff values determined using the 95th percentile of women who visited the clinic. To determine the cutoff value, 234 women were recruited in 2017. Of these, women who showed outliers were excluded. For outlier rejection, we used the conventional outlier rejection method in which sample values exceeding three standard deviations from the means were rejected. The 95th percentile of TSH was calculated from the remaining 211 women after excluding 23 outliers. Thyroid dysfunction during pregnancy affects the mother, child, and pregnancy outcomes.^7^, ^8^, ^9^ Therefore, 66 women with TSH >3.5 mIU/L who considered levothyroxine (LT4) treatment at the Tawara IVF Clinic were excluded. LT4 has been reported to improve pregnancy outcomes.^16^ Finally, of the remaining 1601 women, 1479 who underwent infertility treatment between March 2015 and February 2018 were enrolled in this study.

### 2.2 Causes of infertility

The causes of female infertility included ovulation disorders (oligo‐ovulation [menstrual cycles longer than 35 days], anovulation, or polycystic ovarian syndrome), disorders involving the fallopian tubes diagnosed with either unilateral or bilateral tubal block using hysterosalpingography (HSG), disorders involving the uterus (leiomyoma, adenomyosis, or endometrial polyps), disorders involving the cervix (abnormal results of at least two Huhner tests), and/or endometriosis. Women requiring infertility treatment because of male infertility, including abnormal results of at least two semen analyses (sperm concentration <15 million/mL or motility <40%) and/or presence of sexual dysfunction, were classified as having male infertility.

### 2.3 Measurements of FT4, TSH, TgAb, and TPOAb

Serum concentrations of TSH (coefficient of variation [CV], 2.7%), FT4 (CV, 2.8%), TgAb, and TPOAb were measured using the electrochemiluminescence immunoassay method with the Elecsys system (Roche) throughout the study period. TSH and FT4 were measured at the Tawara IVF Clinic, and TgAb and TPOAb levels were measured at the Health Sciences Research Institute, Inc. The reference range for FT4 was 0.9‐1.7 ng/dL. For TSH, we used the study‐specific cutoff values of 2.5 and 3.5 mIU/L. Positive TgAb and TPOAb were defined as levels >28 and >16 IU/mL, respectively. TSH, FT4, TgAb, and TPOAb were measured before HSG. During HSG, two types of iodine‐rich contrast media, that is, oil‐soluble contrast medium (OSCM) and water‐soluble contrast medium (WSCM), were used; both are considered risk factors for the development of SCH. We previously reported that the use of WSCM implicated a lower risk for SCH than OSCM.^17^ Thus, WSCM was used in most cases during this study.

Thyroid dysfunction during pregnancy affects the mother, child, and pregnancy outcomes.^7^, ^8^, ^9^ Therefore, in this clinic, patients with TSH >3.5 mIU/L were likely to receive LT4 treatment; because of the effect of LT4 on pregnancy outcomes, these women were excluded from the study (Figure 1).

### 2.4 Cumulative pregnancy and miscarriage rates

In this study, we analyzed non‐assisted reproductive technology (ART) treatment including timed intercourse and artificial insemination of husband's semen (AIH) with/without mild ovary stimulation for 6 cycles and ART treatment for 3 cycles between March 2015 and February 2018. A non‐ART cycle following more than one method of embryonic transfer using ART was excluded from the analysis. Pregnancy was defined as gestational sac detected on ultrasound at a gestational age of approximately 8 weeks. Miscarriage was defined as loss of pregnancy after detection of a gestational sac. Only cases of first pregnancy as a result of infertility treatment were included in the analysis.

### 2.5 Statistical analysis

Results were analyzed using Student's *t* test or Wilcoxon test for comparison between the two groups and Pearson's chi‐square or Fisher's exact test depending on the number of observations in the table cells to compare proportions. We used Kaplan‐Meier curves (log‐rank test) to calculate the cumulative proportion of women who became pregnant in the control and study groups. Furthermore, the miscarriage rate was compared using an adjusted multivariate analysis. Log‐rank test and multivariate analysis were adjusted for age, body mass index (BMI), causes of infertility, TSH, FT4, and prevalence of thyroid antibodies to compare the TSH <2.5 and TSH 2.5‐3.5 groups or adjusted for age, BMI, causes of infertility, TSH, and FT4 to compare the thyroid antibody‐negative groups and TgAb‐positive or TPOAb‐positive groups. *P* < .05 was considered statistically significant. All statistical analyses were performed using R software (R Foundation for Statistical Computing) and JMP9 software (SAS Institute).

## 3 RESULTS

**Figure 2:**
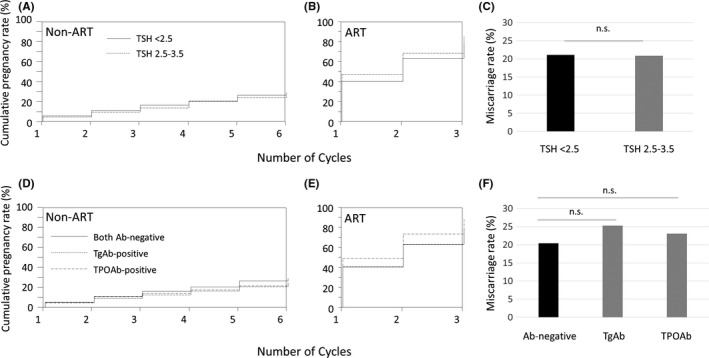
Cumulative pregnancy and miscarriage rates. A‐C, TSH <2.5 group vs TSH 2.5‐3.5 group. D‐F, Thyroid antibody‐negative groups vs TgAb‐positive and TPOAb‐positive groups. Kaplan‐Meier curves for cumulative pregnancy rates of non‐ART (A and D) and ART cycles (B and E) and for miscarriage rates (C and F). Log‐rank test (A, B, D, and E) and multivariate analysis (C and F) after adjusting for age, BMI, causes of infertility, TSH, FT4, and prevalence of thyroid antibodies (A‐C) or adjusting for age, BMI, causes of infertility, TSH, and FT4 (D‐F). TSH, thyroid‐stimulating hormone; TgAb, thyroglobulin antibody; TPOAb, thyroid peroxidase antibody; non‐ART, non‐assisted reproductive technology; ART, assisted reproductive technology; n.s., not significant

This study included 1479 patients (the median self‐reported infertility period was 18 months) who underwent infertility treatment, non‐ART and/or ART, during the study period (Figure 1). There were no differences between the TSH <2.5 and TSH 2.5‐3.5 groups in terms of age, BMI, and history of pregnancy (Table 1). There was no difference in the frequencies of the causes of infertility between the two groups. FT4 was lower and the TgAb‐positive rate was higher in women with TSH 2.5‐3.5 mIU/L (TSH 2.5‐3.5 group) than in those with TSH <2.5 mIU/L (TSH < 2.5 group) (Table 1). Of the 1479 women, 1220 and 755 (ART as the first treatment: n = 259; non‐ART as the first treatment followed by ART: n = 496) underwent treatment with non‐ART and ART, respectively (Figure 1). A comparison of the cumulative pregnancy rate and the miscarriage rate, after controlling for age, BMI, causes of infertility, and prevalence of thyroid antibodies, between the TSH < 2.5 group and the TSH 2.5‐3.5 group was performed (Figure 2A‐C, Table 3, and Table S1). There was no significant difference in the cumulative pregnancy rate between the two groups during the non‐ART (HR, 0.85; 95% CI, 0.56‐1.23) (Figure 2A, Table 3, and Table S1) and ART cycles (HR, 1.17; 95% CI, 0.93‐1.47) (Figure 2B, Table 3, and Table S1). Moreover, there was no difference in the miscarriage rates of the two groups (adjusted odds ratio [AOR], 0.94; 95% CI, 0.56‐1.53) (Figure 2C and Table 3).

**Table 1:** Characteristics of women with TSH levels <2.5 mIU/L and TSH 2.5‐3.5 mIU/L

Patient characteristics	TSH < 2.5 mIU/L (n = 1249)	TSH 2.5‐3.5 mIU/L (n = 230)	*P*‐value
Age^a^	34.3 ± 4.6	34.8 ± 4.6	.11
BMI^a^	20.8 ± 3.1	20.9 ± 3.0	.52
History of pregnancy^b^	0 (0‐8)	0 (0‐5)	.80
History of live birth^b^	0 (0‐3)	0 (0‐2)	.49
History of miscarriage^b^	0 (0‐5)	0 (0‐5)	.97
Thyroid function			
FT4^b^	1.26 ± 0.14	1.22 ± 0.13	<.01
TSH^b^	1.48 ± 0.52	2.91 ± 0.27	<.01
TgAb‐positive^c^	11.5%	17.0%	.03
TPOAb‐positive^c^	9.3%	11.7%	.28
Cause of infertility			
Ovulation dysfunction^c^	7.7%	9.1%	.43
Uterine factors^c^	17.2%	20.0%	.30
Tubal factors^c^	12.8%	9.1%	.13
Cervical factors^c^	12.3%	14.3%	.39
Endometriosis^c^	12.1%	14.3%	.33
Male factors^c^	23.7%	18.3%	.07

Note

Values are denoted as means ± SD or median (min‐max).

Abbreviations: Ab, antibody; BMI, body mass index; FT4, free T4; TgAb, thyroglobulin antibody; TPOAb, thyroid peroxidase antibody; TSH, thyroid‐stimulating hormone.

*P*‐values are based on

a Student's t test,

b Wilcoxon test,

c Chi‐squared test, or Fisher's exact test.

Furthermore, we examined whether the presence of thyroid antibodies contributed to prolongation of the period of treatment to become pregnant (Table 2, Figure 2D‐F, Table 3, and Table S2). The women in the TgAb group were older than those in the thyroid antibody‐negative group, and the BMI was higher in the TPOAb group than in the thyroid antibody‐negative group (Table 2). There were no differences in pregnancy history and cause of infertility between the antibody‐negative group and the TgAb‐positive group or the TPOAb‐positive group (Table 2). In our study, TgAb‐ and TPOAb‐positive women accounted for 12.4% and 9.7% of the subjects, respectively (Table 2); 39.3% of the TgAb‐positive patients also had TPOAb and 50.0% of TPOAb‐positive patients also had TgAb (Table 2). Seventy‐two patients (4.9%) had both antibodies (Table 2). After controlling for age, BMI, causes of infertility, TSH, and FT4, the cumulative pregnancy rates of the TgAb‐ and TPOAb‐positive women were not significantly different from those of the antibody‐negative women. When compared to women without thyroid antibodies, the HR of women with TgAb in the non‐ART group was 0.87 (95% CI, 0.55‐1.32) (Figure 2D, Table 3, and Table S2); HR was 1.09 (95% CI, 0.84‐1.39) for women in the ART group (Figure 2E, Table 3, and Table S2). The HRs of women with TPOAb in the non‐ART and ART groups were 0.88 (95% CI, 0.52‐1.39) (Figure 2D, Table 3, and Table S2) and 1.29 (95% CI, 0.97‐1.68) (Figure 2E, Table 3, and Table S2), respectively. Moreover, there was no significant difference in the miscarriage rates between the antibody‐negative and the TgAb‐ or TPOAb‐positive groups (vs TgAb: AOR, 1.15; 95% CI, 0.67‐1.90; TPOAb: AOR, 1.09; 95% CI, 0.60‐1.91) (Figure 2F).

**Table 2:** Characteristics of women with and without thyroid antibody

Patient characteristic	Ab‐negative (n = 1224)	TgAb* (n = 183, 12.4%)	*P*‐value	TPOAb* (n = 144, 9.7%)	*P*‐value
Age^a^	34.3 ± 4.7	35.0 ± 4.4	.04	34.6 ± 4.5	.38
BMI^a^	20.8 ± 3.0	21.0 ± 3.2	.28	21.7 ± 3.7	<.01
History of pregnancy^b^	0 (0‐8)	0 (0‐7)	.96	0 (0‐5)	.26
History of live birth^b^	0 (0‐3)	0 (0‐2)	.46	0 (0‐2)	.35
History of miscarriage^b^	0 (0‐5)	0 (0‐3)	.23	0 (0‐3)	.89
Thyroid function					
FT4^b^	1.25 ± 0.14	1.25 ± 0.14	.60	1.24 ± 0.15	.32
TSH^b^	1.69 ± 0.70	1.80 ± 0.80	.06	1.73 ± 0.75	.61
TgAb‐positive^c^	0.0%	100.0%	<.01	50.0%	<.01
TPOAb‐positive^c^	0.0%	39.3%	<.01	100.0%	<.01
Cause of infertility					
Ovulation dysfunction^c^	7.8%	9.3%	.47	7.6%	1.00
Uterine factors^c^	17.8%	18.0%	.92	16.0%	.65
Tubal factors^c^	12.4%	12.0%	1.00	11.1%	.79
Cervical factors^c^	12.6%	14.8%	.41	12.5%	1.00
Endometriosis^c^	12.1%	14.2%	.40	12.5%	.89
Male factors^c^	22.8%	26.2%	.30	18.8%	.29

Note

* Seventy‐two patients also had the other antibody (positive for both antibodies).Values are denoted as means ± SD or median (min‐max).Abbreviations: Ab, antibody; BMI, body mass index; FT4, free T4; TgAb, thyroglobulin antibody; TPOAb, thyroid peroxidase antibody; TSH, thyroid‐stimulating hormone.P‐values are based on^a^ Student's t test,^b^ Wilcoxon test,^c^ Chi‐squared test, or Fisher's exact test.

**Table 3:** Adjusted hazard ratios for cumulative pregnancy rates and adjusted odds ratios for miscarriage

	Cumulative pregnancy		Miscarriage
	Non‐ART	ART	
	Adjusted HR (95% CI)		Adjusted OR (95% CI)
TSH < 2.5	1.00	1.00	1.00
TSH 2.5‐3.5	0.85 (0.56‐1.23)	1.17 (0.93‐1.47)	0.94 (0.56‐1.53)
Ab‐negative	1.00	1.00	1.00
TgAb	0.87 (0.55‐1.32)	1.09 (0.84‐1.39)	1.15 (0.67‐1.90)
TPOAb	0.88 (0.52‐1.39)	1.29 (0.97‐1.68)	1.09 (0.60‐1.91)

Note

HR and OR were adjusted for age, BMI, causes of infertility, TSH, FT4, and prevalence of thyroid antibodies to compare the TSH <2.5 group and TSH 2.5‐3.5 groups or adjusted for age, BMI, causes of infertility, TSH, and FT4 to compare the thyroid antibody‐negative groups and TgAb‐positive or TPOAb‐positive groups.

Abbreviations: ART, assisted reproductive technology; HR, hazard ratio; Non‐ART, non‐assisted reproductive technology; OR, odds ratio; Ab, antibody; TgAb, thyroglobulin antibody; TPOAb, thyroid peroxidase antibody; TSH, thyroid‐stimulating hormone.

## 4 DISCUSSION

Several investigators have claimed that TSH should be controlled and adjusted to the target level of 2.5 mIU/L in women seeking to become pregnant.^3^, ^4^, ^5^, ^6^, ^10^, ^18^ It has been reported that patients with TSH <2.5 mIU/L have a higher pregnancy rate with either AIH 3 or in vitro fertilization 4 and lower miscarriage rates 18 than those with TSH >2.5 mIU/L. In addition, in patients with TSH >2.5 mIU/L, reports have shown that the pregnancy rate increases and the miscarriage rate decreases by adjusting TSH to <2.5 mIU/L by LT4 treatment.^5^, ^6^ Conversely, other reports have shown no difference in pregnancy, miscarriage, and birth rates between patients with TSH <2.5 mIU/L and those with TSH >2.5 mIU/L.^11^, ^12^, ^13^ In our study, the causes of infertility among women in the TSH 2.5‐3.5 group were not significantly different from those of women in the TSH <2.5 group (Table 1). Furthermore, comparing the TSH 2.5‐3.5 and TSH <2.5 groups, prolongation of the period of treatment required to become pregnant was not observed in the TSH 2.5‐3.5 group (Figure 2A and B, Table 3, and Table S1). Moreover, our study showed no differences in miscarriage rates between the groups (Figure 2C and Table 3).

A possible explanation for the conflicting results obtained using a cutoff value of TSH 2.5 mIU/L is that differences in the dietary iodine intake were dependent on race and diet.^19^ According to a survey conducted in China, TSH levels are higher in coastal residents, who have a higher iodine intake, than in people who live inland and have a lower iodine intake.^20^ Furthermore, TSH reference levels in an iodine‐deficient population have been reported to be low.^21^ These results suggest the possibility that TSH levels that have an adverse effect on reproductive performance are different between races or between populations with different dietary patterns. Our study was conducted in Japan, where iodine intake from edible seaweeds is highest in the world.^22^, ^23^, ^24^ Therefore, basal TSH levels may be higher in Japanese women than in women who live in other areas and do not have sufficient iodine intake. In our study, approximately 15%‐20% of women with normal FT4 had serum TSH levels >2.5 mIU/L. Therefore, a TSH level of 2.5 mIU/L may be a severe cutoff point for defining SCH in Japanese women or in those with sufficient iodine intake who want to become pregnant.

In addition, we examined whether the presence of thyroid antibodies might affect the cumulative pregnancy rate because the presence of thyroid antibodies is associated with the development of SCH.^2^ However, in our study, no significant differences were observed in the duration of infertility treatment and miscarriage rate between TgAb‐ or TPOAb‐positive patients and the antibody‐negative group (Figure 2D‐F, Table 3, and Table S2). These results indicate that the prevalence of thyroid antibodies may not affect the potential conception period among women with TSH <3.5 mIU/L. However, a meta‐analysis showed that the prevalence of thyroid antibodies is associated with miscarriage and preterm birth.^25^ Negro et al conducted a study in 984 pregnant women and concluded that the higher risk of miscarriage observed in TPOAb‐positive women with normal thyroid function was due to the enhancement of SCH during pregnancy.^26^ Thus, although our results showed that mildly elevated TSH (2.5‐3.5 mIU/L) and thyroid antibody positivity did not contribute to prolonged infertility treatment or increased miscarriage rates in thyroid antibody‐positive women, follow‐up testing of thyroid function is important, especially during fertility treatment and early pregnancy.

This study had some limitations. First, we were unable to analyze the effects of controlled thyroid function on the improvement in fertility outcomes among women with TSH >3.5 mIU/L (approximately 5.0% of women). Second, the type of endometrial preparation method for embryo transfer could not be considered in the ART group. This study found no significant differences in the number of treatments required to become pregnant or in the miscarriage rates between women with TSH levels 2.5‐3.5 mIU/L and those with TSH <2.5 mIU/L, irrespective of the presence/absence of thyroid antibodies. We believe that these findings are important for avoiding unnecessary TSH control in women seeking to become pregnant.

## CONFLICT OF INTEREST

Shuhei So and Nao Murabayashi are affiliated with the laboratory of Tawara IVF Clinic, which funded the study. Wakasa Yamaguchi, Naomi Miyano, and Fumiko Tawara declare that they have no conflict interest.

## ETHICAL APPROVAL

The protocol for this study was approved by the Institutional Review Board (IRB) at Tawara IVF Clinic and Hamamatsu University School of Medicine.

## HUMAN RIGHTS STATEMENTS AND INFORMED CONSENT

All procedures followed were in accordance with the ethical standards of the responsible committee on human experimentation (institutional and national) and with the Helsinki Declaration of 1964 and its later amendments. Informed consent was obtained from all patients for inclusion in the study.
