# Supplementary material for: Effect of moderately increased thyroid‐stimulating hormone levels and presence of thyroid antibodies on pregnancy among infertile women
Source: Reprod Med Biol. 2019 Nov 11;19(1):82–8. doi: 10.1002/rmb2.12306 (PMC6955587; doi:10.1002/rmb2.12306)
Supplement: Supplementary file 1 [file RMB2-19-82-s001.docx]

Supplementary Table 1. Life table analysis of cumulative pregnancy rates of non-ART cycles and ART cycles: TSH <2.5 group vs. TSH 2.5-3.5 group

| Non-ART cycles | Groups | Cycle 1 | Cycle 2 | Cycle 3 | Cycle 4 | Cycle 5 | Cycle 6 | ART cycles | Groups | Cycle 1 | Cycle 2 | Cycle 3 |
| --- | --- | --- | --- | --- | --- | --- | --- | --- | --- | --- | --- | --- |
| Patients, n | <2.5 | 1033 | 791 | 627 | 486 | 400 | 297 | Patients, n | <2.5 | 634 | 305 | 128 |
|  | 2.5-3.5 | 187 | 137 | 106 | 84 | 61 | 47 |  | 2.5-3.5 | 121 | 53 | 23 |
| Censors, % | <2.5 | 15.3% | 13.0% | 14.3% | 11.3% | 15.6% | 49.1% | Censors, % | <2.5 | 10.7% | 16.2% | 36.9% |
|  | 2.5-3.5 | 18.3% | 14.9% | 13.8% | 16.8% | 15.3% | 49.5% |  | 2.5-3.5 | 8.3% | 14.5% | 32.4% |
| Average age±SD^†^ | <2.5 | 34.0±4.5 | 34.1±4.4 | 34.0±4.3 | 34.1±4.3 | 34.2±4.3 | 34.3±4.2 | Average age±SD^†^ | <2.5 | 35.1±4.7 | 35.9±4.6 | 36.6±4.3 |
|  | 2.5-3.5 | 34.6±4.7 | 34.8±4.5 | 34.5±4.3 | 34.5±4.5 | 34.7±4.3 | 34.6±4.3 |  | 2.5-3.5 | 35.7±4.3 | 36.7±4.3 | 37.4±4.5 |
| AIH/timing ratio^‡^ | <2.5 | 0.29 | 0.62 | 0.87 | 1.11 | 1.31 | 1.18 | SET, %^‡^  (blast/cleavage ratio^‡^) | <2.5 | 98.7% (0.92) | 97.1% (1.70) | 78.1% (2.00) |
|  | 2.5-3.5 | 0.31 | 0.59 | 0.96 | 1.10 | 1.26 | 1.47 |  | 2.5-3.5 | 100% (0.95) | 98.1% (2.25) | 78.3% (4.00) |
| Pregnancy  per cycle, %^‡‡^ | <2.5 | 5.4% | 5.8% | 5.7% | 4.9% | 7.3% | 3.7% | Pregnancy  per cycle, %^‡‡^ | <2.5 | 39.9% | 38.7% | 41.4% |
|  | 2.5-3.5 | 4.3% | 5.1% | 4.7% | 7.1% | 4.9% | 2.1% |  | 2.5-3.5 | 47.9% | 37.7% | 52.2% |
| Cumulative pregnancy rate, % | <2.5 | 5.4% | 10.9% | 16.0% | 20.2% | 26.0% | 28.7% | Cumulative  pregnancy  rate, % | <2.5 | 39.9% | 63.2% | 78.4% |
|  | 2.5-3.5 | 4.3% | 9.2% | 13.5% | 19.6% | 23.6% | 25.2% |  | 2.5-3.5 | 47.9% | 68.1% | 84.7% |

^†^ Student *t*-test, ^‡^ chi-squared test, or Fisher’s exact test. ^‡‡^Age-adjusted comparison. There was no significant difference between the <2.5 and 2.5-3.5 groups. AIH, artificial insemination of husband; ART, assisted reproductive technology; SET, single embryo transfer

Supplementary Table 2. Life table analysis of cumulative pregnancy rates of non-ART cycles and ART cycles: Thyroid antibody-negative group vs. TgAb-positive group or TPOAb-positive group

| Non-ART cycles | Groups | Cycle 1 | Cycle 2 | Cycle 3 | Cycle 4 | Cycle 5 | Cycle 6 | ART cycles | Groups | Cycle 1 | Cycle 2 | Cycle 3 |
| --- | --- | --- | --- | --- | --- | --- | --- | --- | --- | --- | --- | --- |
| Patients, n | Negative | 1020 | 783 | 627 | 486 | 391 | 292 | Patients, n | Negative | 602 | 289 | 122 |
|  | TgAb | 148^#^ | 110 | 84 | 67 | 57 | 41 |  | TgAb | 110^##^ | 56 | 27 |
|  | TPOAb | 115^#^ | 87 | 62 | 48 | 41 | 31 |  | TPOAb | 81^##^ | 27 | 12 |
| Censors, % | Negative | 15.2% | 12.5% | 14.1% | 12.4% | 15.2% | 49.1% | Censors, % | Negative | 10.3% | 16.2% | 36.8% |
|  | TgAb | 17.3% | 16.0% | 14.3% | 9.5% | 18.6% | 48.8% |  | TgAb | 7.6% | 16.4% | 36.8% |
|  | TPOAb | 16.7% | 17.9% | 16.2% | 9.4% | 16.3% | 49.2% |  | TPOAb | 14.7% | 6.9% | 29.4% |
| Average age±SD^†^ | Negative | 34.0±4.5 | 34.0±4.5 | 34.0±4.4 | 34.1±4.3 | 34.2±4.3 | 34.2±4.2 | Average age±SD^†^ | Negative | 35.1±4.7 | 35.8±4.7 | 36.6±4.5 |
|  | TgAb | 34.8±4.4 | 35.0±4.3^*^ | 35.0±4.1^*^ | 35.1±4.2 | 35.4±4.3^*^ | 35.5±4.3 |  | TgAb | 36.1±4.2^*^ | 37.6±3.5^*^ | 37.7±3.3 |
|  | TPOAb | 34.3±4.6 | 34.6±4.3 | 34.7±4.0 | 34.7±3.9 | 34.8±3.8 | 35.3±3.4 |  | TPOAb | 35.7±4.1 | 35.9±4.2 | 35.8±4.6 |
| AIH/timing ratio^‡^ | Negative | 0.28 | 0.60 | 0.86 | 1.08 | 1.30 | 1.25 | SET, %^‡^  (blast/cleavage ratio^‡^) | Negative | 98.7% (0.97) | 96.5% (1.79) | 75.4% (1.84) |
|  | TgAb | 0.38 | 0.77 | 1.05 | 1.16 | 1.28 | 0.95 |  | TgAb | 100% (0.83) | 100% (1.33) | 95.8% (6.67) |
|  | TPOAb | 0.34 | 0.78 | 1.14 | 2.00 | 1.56 | 0.94 |  | TPOAb | 100% (0.80) | 100% (1.70) | 75.0% (8.00) |
| Pregnancy  per cycle, %^‡‡^ | Negative | 5.3% | 5.6% | 6.1% | 5.4% | 7.4% | 3.4% | Pregnancy  per cycle, %^‡‡^ | Negative | 40.5% | 38.1% | 41.8% |
|  | TgAb | 4.7% | 4.6% | 3.6% | 4.5% | 5.3% | 4.9% |  | TgAb | 40.9% | 37.5% | 41.7% |
|  | TPOAb | 4.4% | 6.9% | 3.2% | 4.2% | 4.9% | 3.2% |  | TPOAb | 49.4% | 48.2% | 58.3% |
| Cumulative  pregnancy rate, % | Negative | 5.3% | 10.6% | 16.0% | 20.5% | 26.4% | 28.9% | Cumulative  pregnancy rate, % | Negative | 40.5% | 63.4% | 78.7% |
|  | TgAb | 4.7% | 9.1% | 12.3% | 16.2% | 20.6% | 24.5% |  | TgAb | 40.9% | 63.1% | 78.5% |
|  | TPOAb | 4.4% | 11.0% | 13.8% | 17.4% | 21.4% | 24.0% |  | TPOAb | 49.4% | 73.8% | 89.1% |

^*^ p<0.05. p values are based on ^†^ Student *t*-test, ^‡^ chi-squared test, or Fisher’s exact test. ^‡‡^Age-adjusted comparison. ^#^ Sixty-three patients and ^##^ thirty-eight patients also had the other antibody (positive for both antibodies). Values of the TgAb group and TPOAb group were compared to those of the thyroid antibody-negative group. AIH, artificial insemination of husband; ART, assisted reproductive technology; SET, single embryo transfer; TgAb, thyroglobulin antibody; TPOAb, thyroid peroxidase antibody
